# Supplementary material for: Effect of Flavorization on Virgin Olive Oil Oxidation and Volatile Profile
Source: Antioxidants (Basel). 2023 Jan 21;12(2):242. doi: 10.3390/antiox12020242 (PMC9952243; doi:10.3390/antiox12020242)
Supplement: Supplementary file 1 [file antioxidants-12-00242-s001.zip › antioxidants-2149499-supplementary.pdf]

Supplementary Information:

Departamento Quimica Analitica

Odour sheet of sensory attributes of virgin and flavorized olive oils

Name of taster: **Date:**

Sample code:

1. Olive fruit (green)
2. Olive fruit (ripe)
3. Other ripe fruits
4. Apple
5. Green
6. Tomato
7. Sweet
8. Artichoke
9. Basil
10. Rosemary
11. Other (specify):
12. Rancid
13. Other sensory defects (specify):

Further comments:

Figure S1. Profile sheet employed for the evaluation of the olive oil samples (VOO, ROO, BOO).

Table S1. Evolution of the main physico-chemical parameters in the three types of olive oils (VOO, ROO, BOO).

**Time (month)**	**Acidity Index** (% m/m expressed in oleic acid)			**Peroxide Value** (meq O_2/ Kg oil)			**K232**			**K270**		
	**VOO**	**ROO**	**BOO**	**VOO**	**ROO**	**BOO**	**VOO**	**ROO**	**BOO**	**VOO**	**ROO**	**BOO**
**1**	0.48	0.65	0.63	7.41	13.00	10.25	1.578	1.778	1.721	0.206	0.183	0.162
**2**	0.50	0.69	0.66	5.73	10.00	8.37	1.667	1.924	1.780	0.218	0.194	0.173
**3**	0.65	0.71	0.66	5.71	9.20	5.99	1.711	2.024	1.792	0.214	0.293	0.184
**4**	0.70	0.78	0.79	5.75	4.50	6.22	1.693	1.950	1.680	0.210	0.278	0.207
**5**	0.73	0.87	0.83	4.2	5.81	8.00	1.679	1.900	1.610	0.216	0.269	0.216
**6**	0.78	0.89	0.84	6.72	8.98	4.47	1.650	2.00	1.590	0.210	0.290	0.210

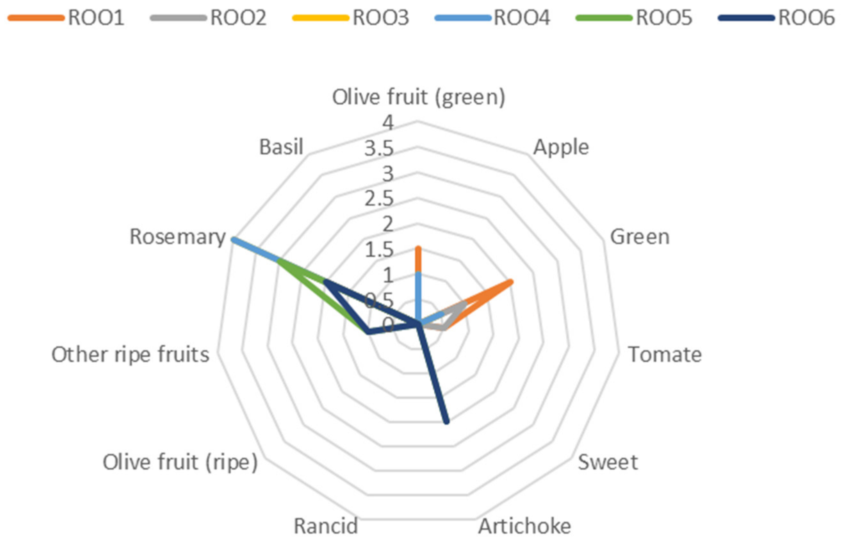

Figure S2. Spider chart evolution of the sensory attributes evaluated during six month stored of rosemary olive oil.

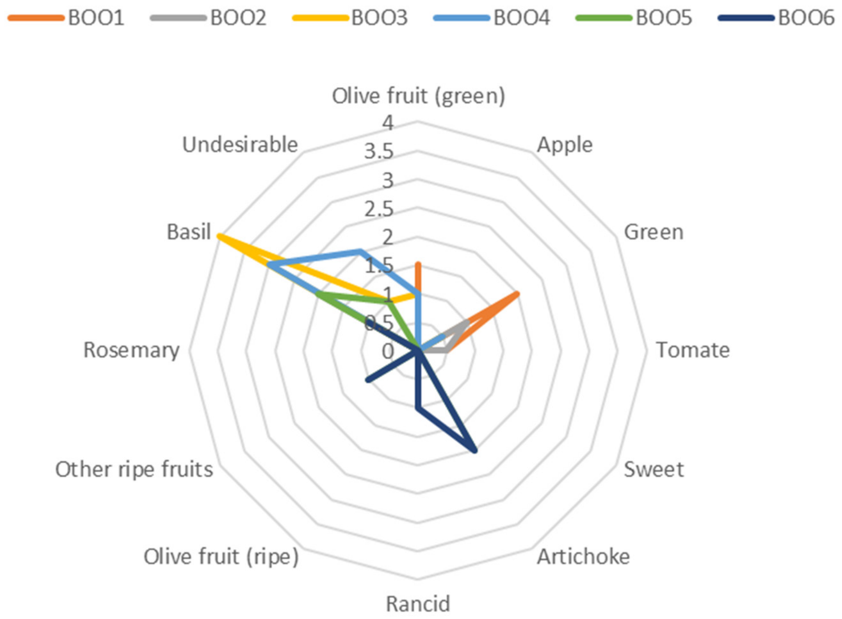

Figure S3. Spider chart evolution of the sensory attributes evaluated during six month stored of basil olive oil.

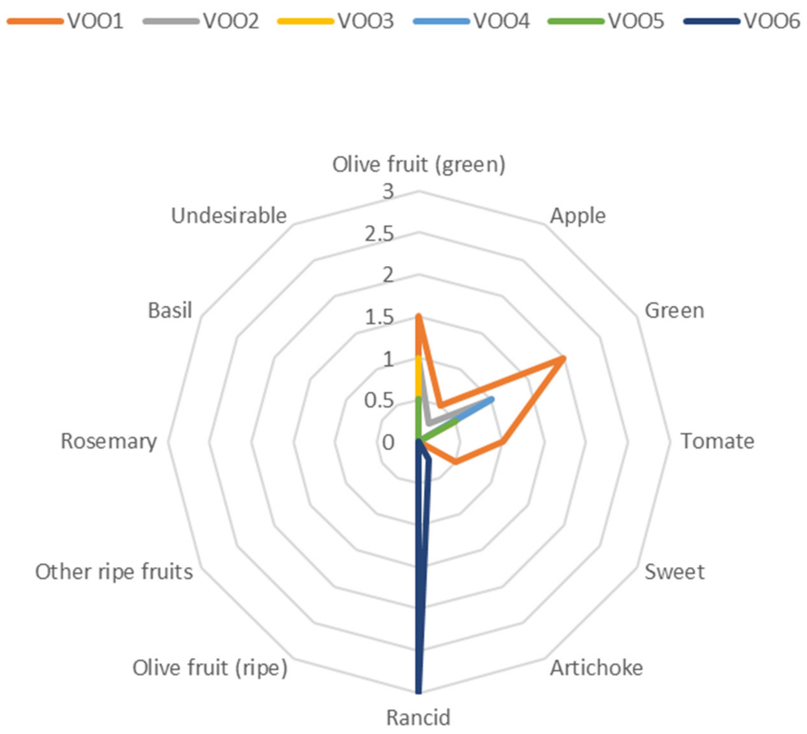

Figure S4. Spider chart evolution of the sensory attributes evaluated during six month stored of virgin olive oil.
